# Decision Tree-Based Sensitive Information Identification and Encrypted Transmission System

**DOI:** 10.3390/e22020192

**Published:** 2020-02-07

**Authors:** Shuang Liu, Ziheng Yang, Yi Li, Shuiqing Wang

**Affiliations:** Electronic Engineering College, Heilongjiang University, Harbin 150080, China; shadows79@163.com (S.L.); 15554201902@163.com (Y.L.); 17863934828@163.com (S.W.)

**Keywords:** decision tree, hardware encryption, ZYNQ, SM4

## Abstract

With the advent of the information age, the effective identification of sensitive information and the leakage of sensitive information during the transmission process are becoming increasingly serious issues. We designed a sensitive information recognition and encryption transmission system based on a decision tree. By training sensitive data to build a decision tree, unknown data can be classified and identified. The identified sensitive information can be marked and encrypted to achieve intelligent recognition and protection of sensitive information. This lays the foundation for the development of an information recognition and encryption transmission system.

## 1. Introduction

Encryption technology is an important measure for information security. Therefore, this paper adopts FPGA (Field Programmable Gate Array) chip hardware to implement a domestic SM4 (A block cipher standard adopted by the government of the People’s Republic of China) encryption algorithm, and proposes a hardware encryption transmission design scheme based on a fully programmable SOC (System on Chip) platform [1,2]. Furthermore, we make use of an FPGA and ARM (Advanced RISC Machine) processor co-design to enhance the practicability and extensibility of the encryption system. In the FPGA development environment, the PCIe (Peripheral Component Interconnect express) interface design is implemented with the IPI (Intellectual Property Integrator) tool to perform high-speed data transmission between the sending end and the FPGA board card [3]. The Verilog HDL (Hardware Description Language) language is used to implement the data encryption and decryption operations of the SM4 block cipher algorithm, which is encapsulated into an independent encrypted IP (Intellectual Property) core and called in the system [4]. In this paper, a face recognition technique using a decision tree is proposed to encrypt the matched faces.

When receiving the photos of people who need to be queried, the system automatically reads the camera information and extracts the people, so as to complete face recognition. After identifying important people, we add their tag information and encrypt the transmission with the hardware; this achieves the effect of intelligent recognition and the protection of sensitive information.

The information security problem is not only related to the personal privacy problem, it is also related to business interests, and the national security problem; therefore, how to ensure information security becomes an important problem that must be solved in the new era of development in this sector. By implementing the encryption algorithms through FPGA, provides a set of solutions for hardware encryption of important information for network information security. In the field of communications, with the improvement of bandwidth and performance requirements, traditional buses have been gradually phased out. At this time, the PCIe bus as a third-generation interconnect bus successfully solved the bottleneck problem during high-speed data transmission and obtained good applications [5,6], which also means that the era when the traditional parallel bus started to develop towards the high-speed serial bus has arrived. The performance of the PCIe bus is excellent. It supports point-to-point serial connections between chips and devices and is characterized by a low overhead and low latency, thus greatly improving the bandwidth of the effective bus data [7,8]. In high-speed data transmission and storage systems, transmission through the PCIe bus has become the first choice. In this design, high-speed data transmission is also required for encryption and decryption. Therefore, the PCIe interface on the FPGA board card was developed to realize high-speed communication between the transmitter and the FPGA by using its DMA (Direct Memory Access) function.

Software encryption has the advantages of simple implementation, low cost, and convenient updating, but software encryption occupies a large amount of CPU resources and causes system stalls. Additionally, it is also easy for attackers to use analysis programs to track, decompile and other means to attack [9], it has poor security, key management is more complex, and implementation is difficult. Hardware encryption is mainly implemented by a special encryption card or FPGA, with a fast encryption and decryption speed, a relatively complicated encryption process, and protection against being tracked by the outside world and attacked [10,11], as well as it being difficult to crack the hardware; therefore, the security reliability of hardware encryption is relatively high, which also means hardware encryption is being adopted by more and more enterprises.

A decision tree is a simple but widely used classifier.

By constructing the decision tree with the training data, the unknown data can be classified efficiently.

The decision number has two advantages:

● The decision tree model is readable and descriptive, which is helpful for manual analysis;
● It is highly efficient: the decision tree only needs to be constructed once and is used repeatedly; the maximum calculation time of each prediction cannot exceed the depth of the decision tree [12].

## 2. Sensitive Information Identification

### 2.1. Decision Tree

The decision tree algorithm is a method to approximate the value of discrete functions. It is a typical classification method that first processes the data, generating readable rules and decision trees using inductive algorithms, and then it analyzes the new data using the decision [13,14]. In essence, a decision tree is a process of classifying data through a series of rules.

Decision tree algorithms first emerged in the 1960s and late 1970s. The ID3 algorithm was proposed by J Ross Quinlan; it aims to reduce the depth of the tree, but ignores the study on the number of leaves [15]. The C4.5 algorithm was improved on the basis of the ID3 algorithm: the missing value processing, pruning technology, derivation rules, and other aspects of predictive variables are greatly improved, making it suitable for classification problems and regression problems [16,17].

The decision tree algorithm constructs a decision tree to discover classification rules contained in the data. How to construct a decision tree with high precision and a small scale is the core content of a decision tree algorithm.

The classification problem represents the process of classifying instances based on features, which can be considered as an “if-then” set, or as a conditional probability distribution defined on feature space and class space. Decision trees usually have three steps: feature selection, decision tree generation, decision tree pruning [18].

Classification by decision tree: Starting from the root node, a certain feature of the instance is tested, and the instance is assigned to the next node according to the test results. At this time, each child node corresponds to a value of the feature. Test and assign the instance recursively until it reaches the leaf node, and finally divide the instance into the leaf node’s class.

### 2.2. Information Gain

#### 2.2.1. The Meaning of Information Entropy to a Decision Tree

Because there are many bifurcation cases in the process of constructing a decision tree, the decision tree introduces a very important concept: information entropy.

We apply “information entropy” as an index to measure the uncertainty (purity) of the sample set, use the information gain as a measure of purity, and select the feature with the largest information gain to split (as the node).

The larger the information gain, the more concise the tree will be because this property is the root of the tree; therefore, the decision tree construction principle is to choose the maximum information gain.

Intuitively, if a feature has better classification ability, or if the training dataset is divided into subsets according to this feature so that each subset has the best classification under the current conditions, then this feature should be selected more. Information gain is a good representation of this intuitive criterion.

Entropy is defined as the expected value of information. If the thing to be classified may be divided into multiple classes, the information entropy of the symbol $x_{i}$, is defined as

(1) $$l\left(x_{i}\right)=-log_{2}p\left(x_{i}\right)$$

where $p\left(x_{i}\right)$ is the probability of choosing the category.

In order to calculate entropy, we need to calculate the expected value of the information contained in all possible values of all categories, which can be obtained by the following formula:

(2) $$H=-\overset{n}{\underset{i=1}{\sum }}p\left(x_{i}\right)log_{2}p\left(x_{i}\right)$$

where *n* is the number of classifications, and the greater the entropy, the greater the uncertainty of the random variable.

When the probability in entropy is obtained from data estimates (especially maximum likelihood estimates), the corresponding entropy is called empirical entropy. The empirical entropy formula is as follows:

(3) $$H\left(D\right)=-\sum \frac{\left|c_{k}\right|}{\left|D\right|}log_{2}\frac{\left|c_{k}\right|}{\left|D\right|}$$

Before we get to the information gain, we should clarify what is meant by conditional entropy.

The information gain represents the degree to which the information about feature *X* reduces the information uncertainty of class *Y*.

Conditional entropy *H*(*Y*|*X*) represents the uncertainty of random variable *Y* under the condition that the random variable *X* is known. Conditional entropy *H*(*Y*|*X*) of random variable *Y* under the condition that the random variable *X* is given is defined by

(4) $$H\left(Y|X\right)=\overset{n}{\underset{i=1}{\sum }}p_{i}H(Y|X=x_{i})$$

where $p_{i}=P\left(X=x_{i}\right)$ when the probability of entropy and conditional entropy is obtained by data estimation (especially maximum likelihood estimation); the corresponding values are empirical entropy and empirical conditional entropy, respectively. In this case, if there is 0 probability, then 0log0=0.

#### 2.2.2. Information Gain

Information gain is relative to the feature. Therefore, the information gain *g*(*D*, *A*) of feature *A* to the training dataset *D* is defined as the difference between the empirical entropy *H*(*D*) of set *D* and the empirical entropy *H*(*D*|*A*) of *D* under the given conditions of feature *A*, namely,

(5) g(D,A)=H(D)−H(D|A)

Generally, the difference between entropy *H*(*D*) and conditional entropy *H*(*D*|*A*) becomes mutual information. The information gain in decision tree learning is equivalent to the mutual information of classes and features in the training dataset.

The size of the information gain value is relative to the training dataset, and it has no absolute significance. When the classification problem is difficult, that is, when the empirical entropy of the training dataset is large, the information gain value is large, otherwise the information gain value is small. You can use the information gain ratio to correct this problem, which is another criterion for feature selection.

Information gain ratio: the information gain ratio $g_{R}\left(D,A\right)$ of feature A to training dataset D is defined as the ratio of its information gain g(D,A) to the empirical entropy of training dataset *D*:

(6) $$g_{R}\left(D,A\right)=\frac{g\left(D,A\right)}{H\left(D\right)}$$

### 2.3. Constructing a Classifier with Feature Faces

At the core of the eigenface is an unsupervised dimensionality reduction technique called principal component analysis (PCA). We demonstrate how to apply this general technology to face recognition.

Face recognition is the challenge of classifying faces from the input image. A simple way is to take a new image, flatten it into a vector, and calculate the Euclidean distance between it and all other flattened images in the database.

If we have a large face database, then the comparison will take a while for each face. The larger our dataset, the slower our algorithm. However, more faces also produce better results. We want a system that is both fast and accurate. We want to take high-dimensional images and compress them to smaller dimensions, while preserving the essence or important parts of the image.

Dimensionality reduction is unsupervised learning. We want to take high-dimensional data (such as images) and represent them in low-dimensional space.

In the simple 2D case, we want to find a line to project the points onto. After projecting the points, we will have the data in 1D instead of 2D. Similarly, if we have 3D data, we want to find a plane to project these points down to reduce the dimension of the data from 3D to 2D.

#### 2.3.1. Principal Component Analysis

Principal component analysis (PCA) is a multivariate statistical analysis method in which a number of variables are transformed by a linear transformation to select a small number of important variables.

The core steps involved in PCA selecting these features are as follows:

1. Zero averaging: take the mean of each column, and then subtract the mean from all the numbers in that column;
2. Find the covariance matrix of this matrix;
3. Evaluate the eigenvalue and eigenmatrix;
4. Retain the main components (i.e., retain the first n features with large retention values).

In the actual dimensionality reduction, it is often necessary to transform the coordinate axis to cover as much data as possible, that is, to find the position of the largest variance of the data, which often gives the most important information of the data. After we find it, we record the direction as x-axis, then its y-axis is perpendicular to the x-axis.

Therefore, the idea is to find the standard normalization of each element of the original matrix minus its own mean, then find its covariance, and finally find the eigenvalue and eigenvector of the covariance.

#### 2.3.2. Building a Decision Tree with Sklearn

Sklearn.tree provides a decision tree model for solving classification and regression problems.

Now that we have discussed PCA and eigenfaces, we discuss how to write a face recognition algorithm using sklearn. First, we need a dataset. The dataset for this project is the Cropped Yale face dataset, which is an open dataset. Each folder is related to a person and contains multiple photos of that person.

If you want to create your own facial dataset, you will need multiple pictures of each person’s face (from different angles and in different lighting) and real labels. The more types of faces you use, the better your recognizer will be. The easiest way to create a dataset for facial recognition is to create a folder for each person and then put facial images into it. The pictures should all be the same size, and they should be stored in an array of size NumPy (num examples, height, width).

Finally, we divide the dataset into training and test sets.

We must choose the number of components to reduce to, that is, the output dimension (the number of feature vectors to be projected to), and adjust this parameter to try to get the best results.

Now that we have smaller facial expressions, we apply a classifier that uses dimensionality reduction input and generates class labels.

## 3. Encrypted Transmission System

### 3.1. Basic Functions of the SM4 Encryption Algorithm

Aiming at the problem of wireless LAN equipment security, the SM4 algorithm, as the first commercial cipher block algorithm published by China’s cryptography administration, has attracted much attention since its release.

In 2012, the SM4 algorithm was determined to be the national cryptographic industry standard in China. Both the plaintext and key block lengths of the SM4 algorithm are 128 bits, and the key expansion process and encryption and decryption processes use 32 rounds of non-linear iterative operations. The SM4 algorithm is typical Feistel structure, this structure is symmetrical, so the decryption process is very similar to the encryption process, except that the subkeys are used in the reverse order, the decryption subkey is the reverse order of the encryption subkey, and the encrypted and decrypted data is also 128 bits. 

### 3.2. SM4 Encryption Algorithm Encryption and Decryption Process

Both the data packet length and key length of the SM4 block encryption algorithm are 128 bits. In addition, both encryption and decryption are characterized by 32 rounds of iteration, each round of iteration requires the participation of a round key.

**Figure 1:**
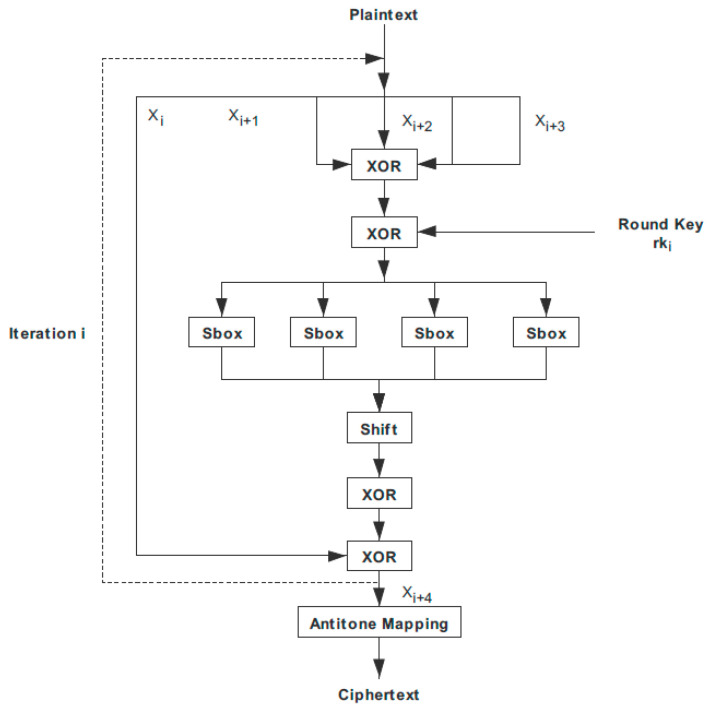
SM4 (A block cipher standard adopted by the government of the People’s Republic of China) encryption implementation block diagram.

Let the input plaintext be (*X*_0_, *X*_1_, *X*_2_, *X*_3_) of 128 bits. The input round key is *rk_i_*, *i* = 0, 1, …, 30, 31, for a total of 32, and the output ciphertext is (*Y*_0_, *Y*_1_, *Y*_2_, *Y*_3_), a total of 128 bits. Then, the encryption algorithm can be expressed as:

(7) $$X_{i+4}=F\left(X_{i},X_{i+1},X_{i+2},X_{i+3},rk_{i}\right)=X_{i}⊕T\left(X_{i+1}⊕X_{i+2}⊕X_{i+3}⊕rk_{i}\right),$$

(8) $$Y_{0},Y_{1},Y_{2},Y_{3}=R\left(X_{32},X_{33},X_{34},X_{35}\right)=\left(X_{35},X_{34},X_{33},X_{32}\right),$$

where the R function is the reverse order output introduced earlier. The decryption algorithm of the SM4 block cipher has the same architecture as the encryption algorithm, except that the 32 rounds of keys used in decryption are used in reverse order. When encrypting, the input order of the round key is *rk*0, *rk*1, *rk*2…, *rk*31. When decrypting, the input order of the round key is *rk*31, *rk*30, *rk*29…, *rk*0. Figure 1 shows the implementation flowchart of the SM4 encryption algorithm. Firstly, 128-bit plaintext or ciphertext is the input, which is divided into four groups after input. The key expansion process produces 32 rounds of subkeys, which participate in the encryption algorithm process through the wheel function. After 32 rounds of iteration, the encryption and decryption operation is completed, and the encrypted ciphertext is output after the reverse sequence operation. If it is a decryption algorithm, only the round key is used in reverse order, and the final output data are also transformed in reverse order, and then combined for output.

The SM4 encryption core is mainly divided into the control module and the function realization module.

The main function of the control module is to identify the type of data frames transmitted and store them accordingly. When a data frame is sent to the protocol analysis module, it is first identified according to the identification flag FFE25D of the frame header (This frame header is selected because it is the byte with the lowest probability in Barker code, which is not easy to be confused with erroneous data and easy to identify). The data frame carries valid data and then receives the data frame header information. The frame header information includes the length of the data and the type of data. The case statement is then used to determine the type of data frame. According to the function of the encryption core, the data frame is divided into three types: key 00, initial plain text 01, and initial cipher text 02. After determining the frame type, enable the corresponding FIFO (First Input First Output) channel to store data, and then wait for the enable signal of the function module for the next operation.

### 3.3. SM4 Encryption IP Core Design

**Figure 2:**
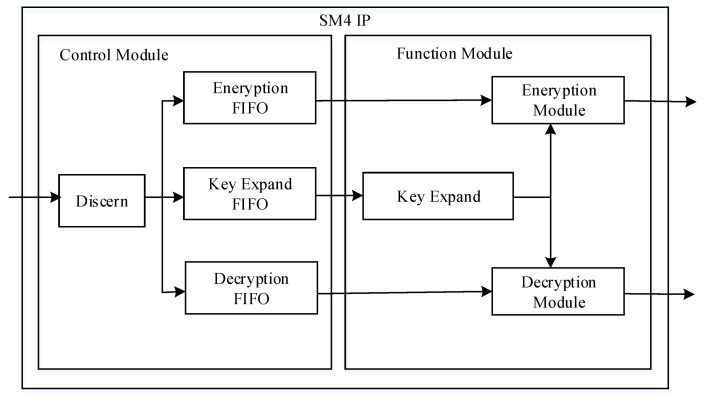
SM4 encryption core internal design block diagram.

The SM4 encryption IP core module is divided into two modules: the control module and function realization module. The internal design block diagram of the encryption module is shown in Figure 2. The control module is divided into the analysis module, the storage module, the function realization module, which is a key expansion module, the encryption module, and the decryption module.

In order to meet the requirements of different functions of the data in the process, the data is first sent to the control module of the encryption core. The protocol analysis module first cages the effective data in FIFO of different categories according to the data’s frame header, and then sends it to the channel of different functions. Because the packet encryption mode is an integer multiple of the packet length, when the data bit width is wrong, FIFO must be used for bit width conversion.

In the function realization module, the key expansion function is first carried out, and the generated subkeys are provided to the encryption and decryption module for the iterative operation. The transmitter first sends the 128-bit initial key to the encryption core through the PCIe interface, expands it into 32 subkeys in the key expansion module, stores the expanded subkeys in the RAM, and sends the expansion completion signal to the encryption and decryption module after the expansion. Encryption module respectively in 32 ciphertext or clear after iterations, the operation mode of the encryption module adopts full-duplex, encryption and decryption module for the mutual interference of two separate modules, can add the declassified work at the same time, but the decryption key is needed when used in reverse order, use the first wheel key is extended when completing the final is the key, so it is worth noting that the work in the encryption module must be completed before the key extension module, the key extension module can make complete signals, then add the decryption operation.

### 3.4. Hardware Implementation

#### 3.4.1. PL-Side Design and Implementation

In the basic configuration of the XDMA (Xilinx DMA IP core) module, we use the X4 link channel design. The transmission method of DMA is AXI(Advanced eXtensible Interface) memory mapped. The bandwidth of a single channel can reach 5GT/s. The AXI4 interface is selected. The data bus bit width is set to 128 bits and 100 MHz for the PCIe (Peripheral Component Interconnect express) reference clock; the 125 MHz AXI interface clock provides clocks for other design modules on the PL (Progarmmable Logic) side. The IP core can be configured with eight independent DMA channels, comprising four H2C channels and four C2H channels. Through these channels, read and write transaction packets can be generated to the PCIe integrated module or host computer.

**Figure 3:**
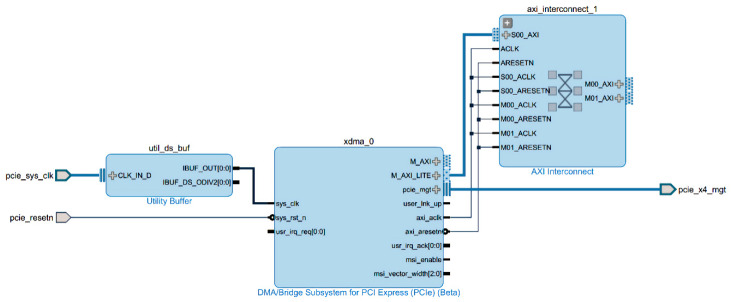
PL (Progarmmable Logic)-side PCIe (Peripheral Component Interconnect express) module design diagram.

For the setting of the clock module, first, the differential clock is connected to the utility buffer IP through the pcie sys_clk external interface, and the reference clock frequency is set to 100 MHz. We use XDMA’s sys_clk interface to input the clock signals to allow it to generate its own internal clock and provide external output clock signals. The clock domain of AXI4 is output from the axi_aclk clock interface on the other end of IP. Other IP cores with AXI interfaces on the PL-side provide the clock. The PL (Progarmmable Logic)-side PCIe (Peripheral Component Interconnect express) module design diagram is shown in Figure 3.

**Figure 4:**
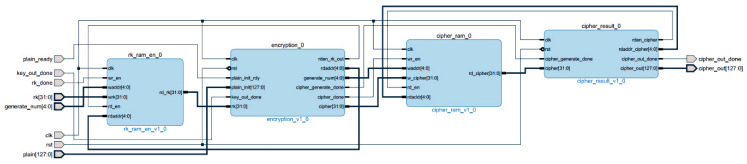
Block diagram of the encryption module.

The internal block diagram of the encryption module is shown in Figure 4. The encryption module reads the 32 wheel keys from the subkey storage module rk_ram_en and participates in the iterative operation of the encryption module encryption. The 32 encrypted sub-ciphertexts are stored in the sub-ciphertext storage module cipher_ram. The final ciphertext synthesis module cipher_result reads the last four rounds of sub-ciphertext, and reverses them into the final ciphertext data.

**Figure 5:**
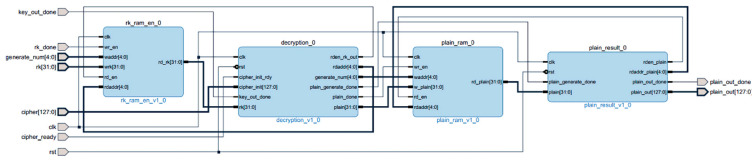
Decryption module internal block diagram.

The internal block diagram of the decryption module is shown in Figure 5. The decryption module reads the 32 wheel keys from the subkey storage module rk_ram_en and participates in the iterative operation of the decryption module decryption. The 32 decrypted child plaintexts are stored in plain_ram, and the final plaintext synthesis module plain_result reads the last four wheels of plaintext and reverses them into the final plaintext data.

**Figure 6:**
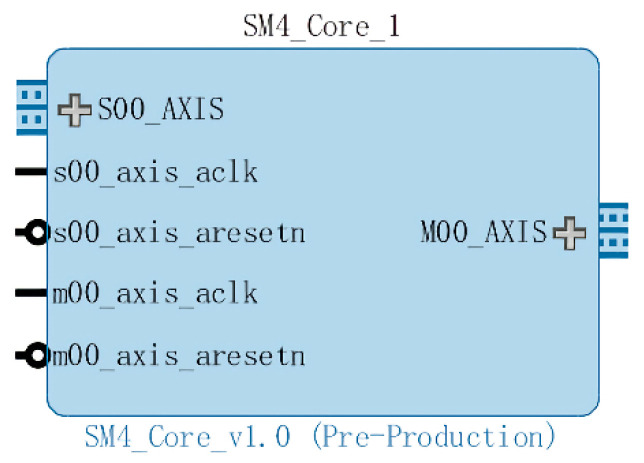
AXI (Advanced eXtensible Interface) interface SM4 encryption IP core.

After we implement the two major modules of control and function in the encryption core, the last thing to do is to encapsulate the SM4 encryption module into a complete IP core, as shown in Figure 6, and call it in the system design. This is because inter-communication uses the AXI bus protocol, so we encapsulate the encryption core into a module with an AXI interface and call it in the system.

After encapsulating the IP, we select the repository manager in the IP settings to add its IP project address, and then we can call our IP in the user repository in the IP catalog.

#### 3.4.2. Communication between PL (Progarmmable Logic) and PS (Processing System)

When the plaintext data to be encrypted is transmitted from the PCIe interface on the PL-side to the SM4 encrypted IP core for encryption, the processed data needs to be stored because the IP of the PCIe and SM4 encryption cores mainly use the AXI interface clock derived from XDMA to work. The clock is 125 MHZ, and the PS-side is derived from the IP core of ZYNQ (Industry’s First Scalable Processing Platform from Xilinx) as 100 MHZ. Therefore, when the PL-side wants to communicate with the PS-side, it needs to process across the clock domain. In order to meet this condition and store the data, it uses the design concept of asynchronous FIFO.

The encrypted data output from the encryption core is buffered in the FIFO. After the PS-end is enabled, the data is read from the FIFO. The main interface of the FIFO is connected to the slave interface of the AXI DMA. The main function of AXI DMA is to provide high-bandwidth direct memory access between memory and AXI interface peripherals. It has a complete on-chip DMA transfer function, which can free the PS (Processing System)-side CPU from the data transfer task. In ZYNQ, AXI DMA is the bridge for FPGA to access DDR3 (Third-generation double-rate synchronous dynamic random access memory), but this process is monitored and managed by ARM (Advanced RISC Machine).

**Figure 7:**
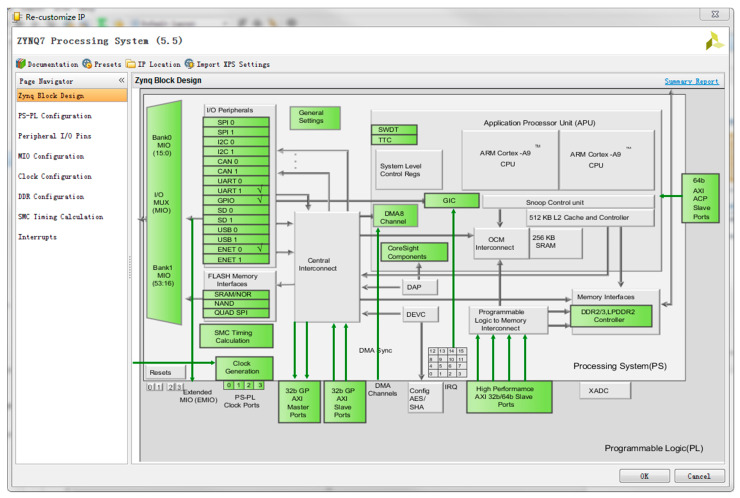
ZYNQ (Industry’s First Scalable Processing Platform from Xilinx) IP (Intellectual property) core setting peripherals.

The data transmitted through AXI_DMA is connected to the ZYNQ processor through the AXI connector. For the ARM processing core, we need to define some settings. The AXI_HP interface on ZYNQ is a high-speed interface with high-performance transmission. The PL module can be connected as a master device, which is mainly used by the PL to access the memory on the PS. We use this interface here to connect PS-side DDR for high-speed encrypted data storage transition. We first need to enable the HP interface in the processor IP. When the data transfer is complete, we also need to enable the interrupt interface and notify the ARM-side to perform the corresponding processing. Later, on the PS-side, the GPIO is used to transmit instructions on the ARM-side and use the network port to send data to the Internet. Therefore, we need to enable the GPIO interface and Ethernet interface in the processor IP. The situation of enabling peripherals in ZYNQ is shown in Figure 7.

**Figure 8:**
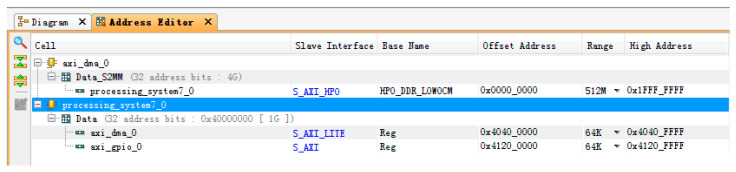
IP core address allocation.

After the IP setting is completed, the next step is to open the address setting page to assign addresses to each IP core controlled by the ARM-side, as shown in Figure 8.

**Figure 9:**
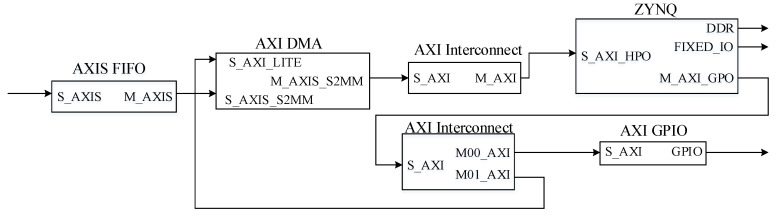
PL and PS communication hardware block diagram.

After that, we only need to synthesize and implement the built hardware system, and finally generate a bit stream file and import the hardware system into the SDK (Software Development Kit). The next step is to perform the bare-metal program of the PL and PS data communication and network port control module in the SDK design. The frame diagram of the PS-end system design is shown in Figure 9.

## 4. Results

### 4.1. Face Recognition

The dataset used in this project is tailored to the Yale facial dataset (Cropped Yale face dataset); it is an open dataset, each folder is associated with a person, and contains more than the person’s photo. There are 38 unique faces in the dataset, each with a maximum of 64 photos. The dataset is a kind of high-dimensional data, and we reduced the dimension.

In this project, we chose to use principal component analysis (PCA), because PCA uses eigenvectors/eigenvalues to selectively identify important features that fully represent the image. Therefore, the first step is to find the characteristics of the face.

**Figure 10:**
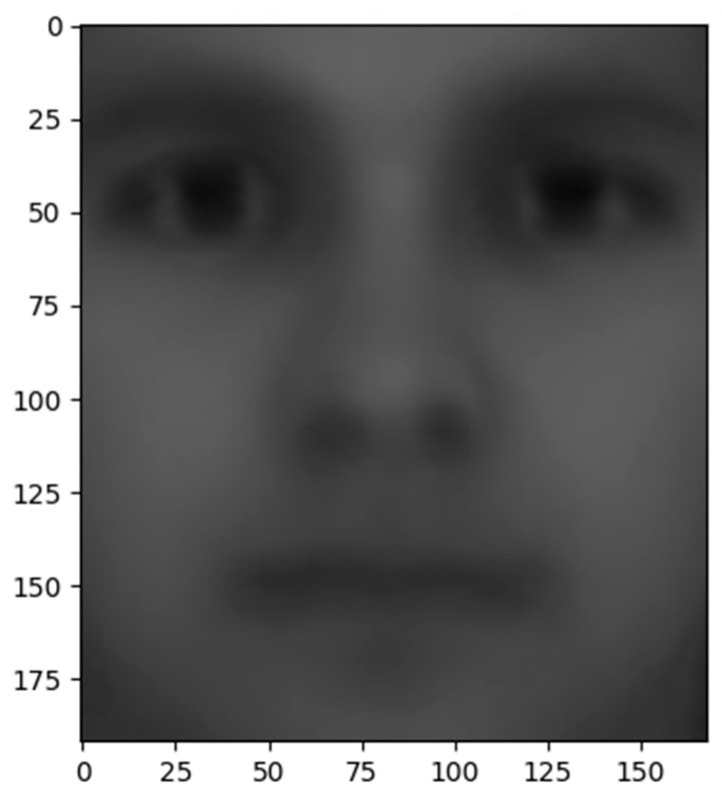
The mean face of the dataset.

In order to find the characteristics of the face, a gradual process is followed:

1. Construct a data matrix D;
2. Find a mean face (like Figure 10);
3. Reduce the dimension of the data matrix.

A dimensionless data matrix means subtracting the mean from each row to ensure that the mean of any column of D is 0;

4. Analyze the obtained matrix using principal component analysis to identify the featured face.

**Figure 11:**
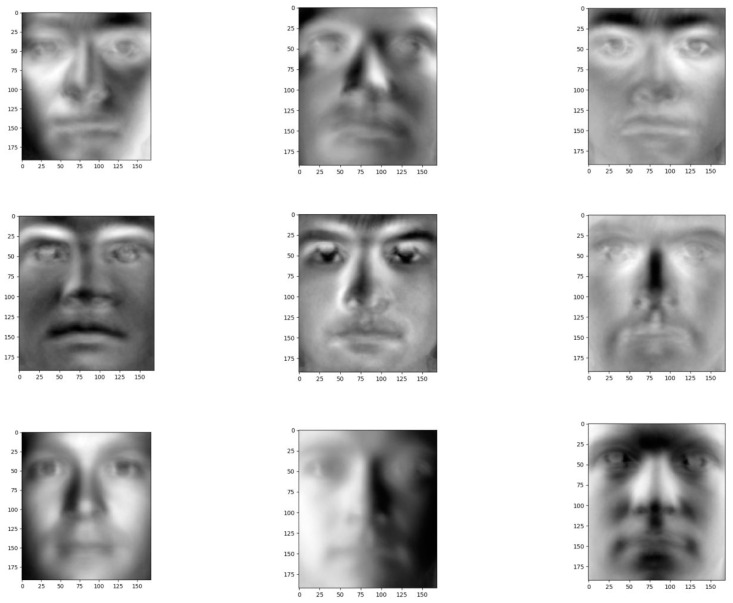
The characteristic surfaces of the dataset.

Plot the first nine principal components, and again reshape each W * H principal component into a w-width and h-height image. These principal components are called characteristic surfaces is shown in Figure 11. We call this the E matrix, and it stores the principal components as rows.

● Data matrix structure.
  1. Load the dataset in grayscale format;
  2. Convert each image into vector format;
  3. These vector horizontal stacked into a matrix *D*, if the width and height of each image *W* and *H*, respectively, the total number of images for *N*, is the size of the *D* for *N* * (*W* * *H*);
  4. Each data point is labeled as the person’s identity, which is stored in an *N*-dimensional vector *Y*.

Reduced data is obtained by projecting the data points onto the principal components by matrix multiplication i.e.,

(9) $$P=D\cdot E^{T}.$$

This means that projections of a data point onto the principal components are obtained by taking the dot product of the data point with each of the principal components. The matrix multiplication just expresses this in a compact and computationally efficient format.

Therefore, the ith row of *P* consists of principal component projections for the ith data point i.e., principal component scores that can be used to reconstruct the ith data point using the principal components in *E*. The jth column of *P* captures the projection of data points onto the jth principal component.

Therefore, by applying PCA, we found the reduced form for all the data points represented using the first nine principal components.

● Building a classification system.

Now, we use the decision trees to build a face classification system. This can be done using the original features as well as the features obtained from PCA-based dimensionality reduction *P*. The ith row in both matrices corresponds to the ith data point. The label for each data point is the person’s identity that was stored into an *N*-dimensional vector.

For the purpose of cross-validation, we split the data (*D*, *P*, and *y*) into 80% training data and 20% test data.

● Fitting a decision tree model to original data.

Initially, we fit a decision tree model using the training data *D* and y without any pruning, that is, we let the tree grow until it is impossible to split (all records in the data subset have the same output or the same set of input attributes).

We use this model to predict the test set y. These predictive labels are evaluated against real labels to calculate test errors.

● Tree pruning.

Pruning is one of the methods used to stop branching of the decision tree. There are two types of pruning: pre-pruning and post-pruning. Pre-pruning is used to set an index during the growth of the tree, and stop the growth when the index is reached. Doing so easily results in a “horizon limit”, that is, once the branch is stopped and node N becomes a leaf node, its successor is severed. These stopped branches will mislead the learning algorithm, resulting in a greatly reduced purity of the generated tree, closer to the root node. The tree, during post-pruning, must first be fully grown until the leaf nodes have the smallest impurity value, so the “horizontal limitation” can be overcome. Then, whether to eliminate them for all adjacent pairs of leaf nodes is considered. If the elimination can cause satisfactory impure growth, then the elimination is performed and their common parent node is made into a new leaf node. This method of “merging” leaf nodes is exactly the opposite of the process of node branching. After pruning, leaf nodes are often distributed on a wide level, and the tree becomes unbalanced. The advantage of post-pruning technology is that it overcomes the “horizontal limitation” effect, and does not need to retain samples for cross-validation, making full use of the information of the entire training set. However, the computational cost of post-pruning is much greater than that of pre-pruning, especially in large sample sets. However, for small samples, post-pruning is still better than pre-pruning. Here, we describe the method of post-pruning.

The purpose of pruning is not to minimize the loss function. The purpose of pruning is to achieve a better generalization ability. For a decision tree, the larger the number of leaf nodes, the more the decision tree responds to the details of the training data, which weakens the generalization ability. To improve the generalization ability, pruning is needed.

● Fitting a decision tree model using the principal component training data P and y.

At the beginning, we fit a decision tree model using the training data P and y without any pruning.We use this model to predict the test set y. These predicted labels are evaluated against the true labels to compute the test error. The number of terminal nodes is 1783. The maximum depth is 29. The number of leaf nodes is 896.

Since the tree is a bit too large, pruning has to be done. In order to fit a pruned tree, the maximum depth hyperparameter is chosen using 10-fold cross-validation (CV).

This pruned tree is evaluated on the test data on the basis of the test error.

**Figure 12:**
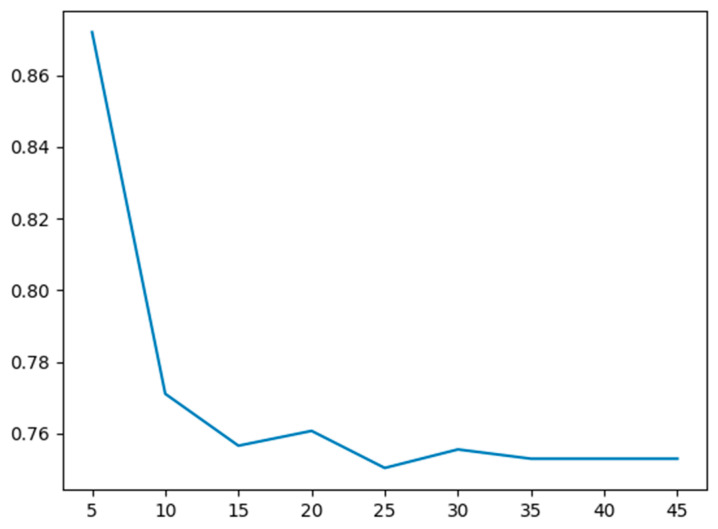
The maximum depth hyperparameter versus the cross-validation error.

The maximum depth hyperparameter versus the cross-validation error is plotted to find the optimal hyperparameter in Figure 12. The test error is about 0.658.

● 2D hyperparameter search.

Now, we not only adjust the maximum tree depth but also performed the 2D cross-validation on the training data P and y to adjust the two hyperparameters (1—the maximum tree depth in the decision tree training; 2—the number of top principal components used as features). A heat map where the X-axis is the maximum tree depth superparameter and the Y-axis is the top principal component characteristic number was drawn.

**Figure 13:**
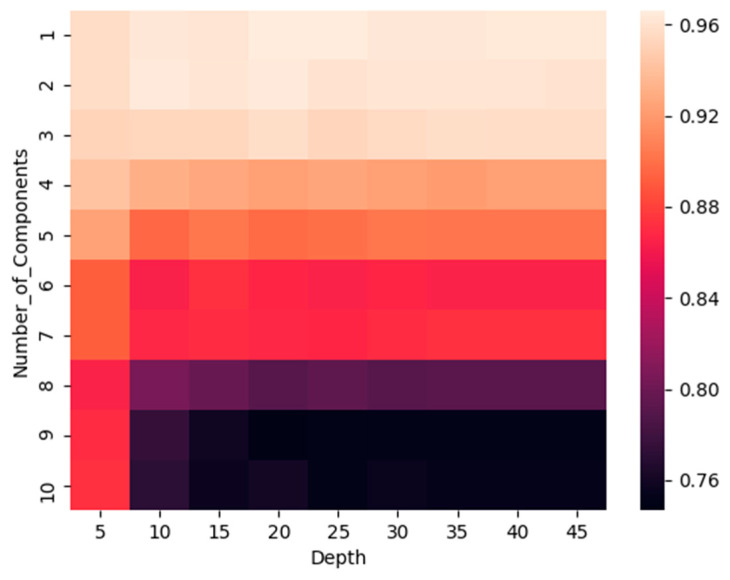
Heatmap wherein the heat value is the cross-validation error. The combination of hyperparameters that minimizes cross-validation error is computed using this heatmap.

The calorific value is a cross-validation error. We use this heat map in Figure 13 to calculate a hyperparameter combination that minimizes cross-validation errors.

● The above model is used to fit the decision tree model.

Using the optimal combination of the hyperparameters that we find, we train the model with the whole training set. Using this model, we predict y in the test set and evaluate these predictions against the real label y to calculate the test error.

The error of the parameter test obtained by the 2D hyperparameter search is about 0.36, which is half of the original value. Obviously, compared with a one-dimensional superparameter search for the depth of the largest tree, a two-dimensional hyperparameter search can help improve the test error.

By projecting the unknown face onto the feature vector, we can see the difference between the unknown face and the average image in different directions. Therefore, if we do this with each row of the bias matrix that we obtained, each row is going to give us a weight vector. We used the Euclidean distance of Ω and $\Omega_{k}$ to judge the difference between the unknown face and the kth trained face.

The smallest k chosen is the face of the training set corresponding to the face. In fact, usually, the distance threshold is set, and when the distance is less than the threshold, it means that the face to be identified is the same person as the KTH face in the training set. When the traversal of all training sets is greater than the threshold value, it can be divided into one of two things: a new face or non-face, according to the size of the distance value. The threshold setting is not fixed according to the training set.

Finally, we can make predictions and use functions to print a complete quality report for each class, as shown in Table 1.

We saw precision, recall, F1-score, and support. Support is only the number of times this ground truth label appears in our test set. In fact, F1-score is calculated based on the precision and recall scores. Precision and recall are more specific indicators than a single accuracy score; the higher the value, the better.

### 4.2. Encryption Transmission

In the overall system design, it was necessary to use to the FPGA and ARM platform. In order to satisfy this, the design chip was selected using the Xilinx company Zynq-7000 series, Zynq-7000 series based on the Xilinx fully programmable extensible processing platform structure. The structure of the processor development platform (hereinafter referred to as the PS-side) was based on the ARM dual-core Cortex-A9 processor. The structure of programmable logic design side (hereinafter referred to as PL) was based on Xilinx programmable logic resources for development.

This system was designed on the basis of the SOC platform, and the PL-end and PS-end were jointly used in the development. This ensured the practicability and extensibility of the encryption system.

**Figure 14:**
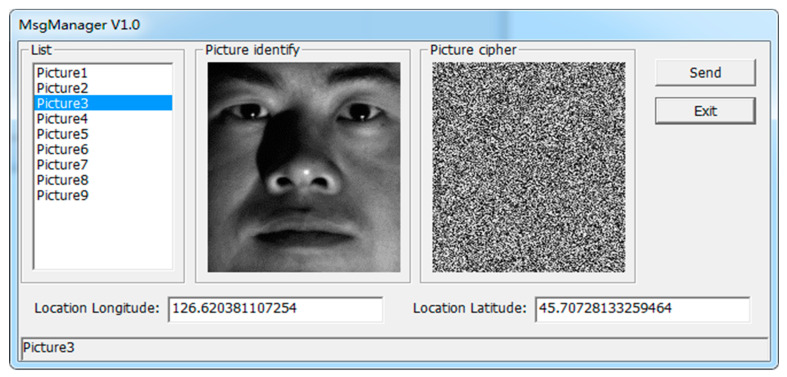
The encryption of sensitive information and additional location information.

One of the faces from the Cropped Yale Face dataset for the encrypted transmission test was selected, as shown in Figure 14.

The performance of this high-speed PCIe encrypted transmission card was compared with that of similar encrypted transmission cards, as shown in Table 2.

The PCIe encryption card in SG DMA mode used in this design has an obvious advantage in data transmission speed compared with similar encryption cards: when the data volume reaches about 1M, the transmission speed can reach 1 GB/s.

## 5. Conclusions and Future Work

Since the tree was a bit too large, pruning had to be done. In order to fit a pruned tree, the maximum depth hyperparameter is chosen using 10-fold cross-validation (CV).

The pruned tree was evaluated using the test data on the basis of the test error.

In view of the increasingly serious information security problem, this paper presents a hardware encryption transmission card, which realizes face recognition using a decision tree, and performs encryption and decryption functions based on an FPGA chip, which has a high level of security.

This system adopts the Xilinx company’s Zynq-7000 series chip, under the fully programmable SOC architecture, utilizing the flexibility of the FPGA and ARM processor co-design, and adopts the IPI tool for development and design, which improves the practicability and extensibility of the encryption system.

This paper is an in-depth study of the hardware design of high-speed PCIe encryption cards. The specific contents are as follows:

1. The construction of a classifier using characteristic faces to achieve face recognition;
2. The maximum depth hyperparameter versus the cross-validation error is plotted to find the optimal hyperparameter;
3. The parameters are obtained through a 2D hyperparameter search. Clearly, the 2D hyperparameter search helped improve test error compared to the 1D hyperparameter search for maximum tree depth;
4. Hardware implementation of each module design of the encryption transmission system. On the FPGA-side, the PCIe interface is developed based on XDMA. The hardware is realized, and high-speed data transmission between host and board is completed. In the SM4 encryption core module, the control module implements protocol parsing and storage, while the function module implements key expansion, encryption, and decryption, and encapsulates this into an IP core with an AXI interface, naming it in the system. Under the ARM processor environment, the DMA data communication between the PL-end and PS-end is realized, and the MAC network control module is used to realize data transmission;
5. Hardware testing is carried out on the sensitive information recognition and encryption transmission system. Firstly, the system test hardware platform was built, and then the function and performance of the PCIe interface were tested and analyzed.

After that, the function of the SM4 encryption core was tested. Finally, the whole encryption transmission system was tested, and the whole system had a complete set of functions.

There are still many areas to be optimized in this design:

1. Although the decision tree is used to realize face recognition, the algorithm is rarely improved, and the recognition accuracy needs to be improved;
2. In the future, we will create and train our own datasets and realize real-time face recognition on video frames;
3. The implementation scheme of the SM4 encryption algorithm can be improved by converting it into an assembly line design; this will increase the total resource consumption of the design, and improve the running speed of the design and the efficiency of the system implementation;
4. When PCIe transfers data, it can use PIO to transfer the key and DMA to transfer the data according to the address; this no longer requires the design of a frame head resolution module, which improves the usability and scalability of the design;
5. In the future, we will use FPGAs to implement public key encryption algorithms to complete digital signatures, key exchanges, etc. These modules will be combined with our existing SM4 IP.

## Figures and Tables

**Table 1:** Quality report.

	Precision	Recall	F1-Score	Support
YaleB02	0.92	0.72	0.81	50
YaleB03	0.89	0.91	0.90	65
YaleB05	0.87	0.63	0.73	46
Average/total	0.89	0.75	0.81	161

**Table 2:** Horizontal comparison of encryption card performance.

Encryption Card Type	Clock	Speed
PCI encryption card	100 MHz	80 MB/s
PCIe encryption card (BL DMA)	100 MHz	500 MB/s
PCIe encryption card (SG DMA)	100 MHz	1 GB/s

## References

[1] Wei H., Cui H., Lu X. (2012). Differential algebraic analysis of the SMS4 block cipher algorithm. J. Chengdu Univ. (Nat. Sci. Ed.).

[2] Cai Y., Qu Y. (2016). Research and design of SMS4 encryption chip based on single-wheel loop structure. Electron. Des. Eng..

[3] Wang Z., Gao Q. (2008). Design of DMA controller based on the FPGA PCIe bus interface. Electron. Technol. Appl..

[4] Li L., Liu B., Wang H. (2016). QTL: A new ultra-lightweight block cipher. Microprocess. Microsyst..

[5] Martínez-Herrera A., Mancillas-López C., Mex-Perera C. (2016). GCM implementations of Camellia-128 and SMS4 by optimizing the polynomial multiplier. Microprocess. Microsyst..

[6] Wu J. (2013). Research and implementation of mixed cryptography algorithm based on SM4 and SM2. Softw. Guide.

[7] Lang H., Zhang L., Wu W. (2008). Fast software implementation technology of SM4. J. Univ. Chin. Acad. Sci..

[8] Wang Y. (2015). Research on the hardware implementation of SMS4 cryptography. Autom. Instrum..

[9] Legat U., Biasizzo A., Novak F. (2011). A compact AES core with on-line error-detection for FPGA applications with modest hardware resources. Microprocess. Microsyst..

[10] Wei Y. (2013). Design of PCIe Bus DMA Platform Based on FPGA.

[11] Mohd B., Hayajneh T., Vasilakos A. (2015). A survey on lightweight block ciphers for low-resource devices: Comparative study and open issues. J. Netw. Comput. Appl..

[12] Magerman D.M. Statistical decision-tree models for parsing. Proceedings of the 33rd Annual Meeting on Association for Computational Linguistics.

[13] Freund Y., Mason L. (1999). The alternating decision tree learning algorithm. Icml.

[14] Polat K., Güneş S. (2007). Classification of epileptiform EEG using a hybrid system based on decision tree classifier and fast Fourier transform. Appl. Math. Comput..

[15] Tso G.K.F., Yau K.K.W. (2007). Predicting electricity energy consumption: A comparison of regression analysis, decision tree and neural networks. Energy.

[16] Mulay S.A., Devale P.R., Garje G.V. (2010). Intrusion detection system using support vector machine and decision tree. Int. J. Comput. Appl..

[17] Pal M., Mather P.M. (2003). An assessment of the effectiveness of decision tree methods for land cover classification. Remote Sens. Environ..

[18] Sahinoglu M. (2005). Security meter: A practical decision-tree model to quantify risk. IEEE Secur. Priv..

